# Dysregulated Proinflammatory and Fibrogenic Phenotype of Fibroblasts in Cystic Fibrosis

**DOI:** 10.1371/journal.pone.0064341

**Published:** 2013-05-29

**Authors:** François Huaux, Sabrina Noel, Barbara Dhooghe, Nadtha Panin, Sandra Lo Re, Dominique Lison, Pierre Wallemacq, Etienne Marbaix, Bob J. Scholte, Patrick Lebecque, Teresinha Leal

**Affiliations:** 1 Louvain Centre for Toxicology and Applied Pharmacology (LTAP), Institute of Experimental and Clinical Research (IREC), Université Catholique de Louvain (UCL), Brussels, Belgium; 2 de Duve Institute, Université Catholique de Louvain (UCL), Brussels, Belgium; 3 Cell Biology, Erasmus MC, Rotterdam, The Netherlands; 4 Pediatric Pulmonology, Cliniques Saint Luc, Université Catholique de Louvain (UCL), Brussels, Belgium; French National Centre for Scientific Research, France

## Abstract

Morbi-mortality in cystic fibrosis (CF) is mainly related to chronic lung infection and inflammation, uncontrolled tissue rearrangements and fibrosis, and yet the underlying mechanisms remain largely unknown. We evaluated inflammatory and fibrosis responses to bleomycin in F508del homozygous and wild-type mice, and phenotype of fibroblasts explanted from mouse lungs and skin. The effect of vardenafil, a cGMP-specific phosphodiesterase type 5 inhibitor, was tested *in vivo* and in culture. Responses of proinflammatory and fibrotic markers to bleomycin were enhanced in lungs and skin of CF mice and were prevented by treatment with vardenafil. Purified lung and skin fibroblasts from CF mice proliferated and differentiated into myofibroblasts more prominently and displayed higher sensitivity to growth factors than those recovered from wild-type littermates. Under inflammatory stimulation, mRNA and protein expression of proinflammatory mediators were higher in CF than in wild-type fibroblasts, in which CFTR expression reached similar levels to those observed in other non-epithelial cells, such as macrophages. Increased proinflammatory responses in CF fibroblasts were reduced by half with submicromolar concentrations of vardenafil. Proinflammatory and fibrogenic functions of fibroblasts are upregulated in CF and are reduced by vardenafil. This study provides compelling new support for targeting cGMP signaling pathway in CF pharmacotherapy.

## Introduction

Cystic Fibrosis (CF), an inherited disorder due to mutations of the *CF Transmembrane conductance Regulator* (*CFTR*) gene, has a complex phenotype with multiple clinical manifestations; but lung disease, characterized by chronic airway obstruction, infection and inflammation, accounts for the major cause of morbi-mortality. Fibrosis of the pancreas was recognized since the description of the disease [1]. Extensive pulmonary fibrosis is a common finding in end-stage CF lung disease, and tissue remodelling with increased collagen deposition has been demonstrated in distal airways of young children with CF [2].

Fibroblasts form a heterogeneous population of interstitial stromal cells, the major functions of which are production of extracellular matrix maintaining parenchymal tissue architecture and regulating fibroproliferative repair. However, it has been recognized that fibroblasts may provide much more than a scaffold for parenchymal tissue. They are indeed able to secrete powerful inflammatory chemoattractants, including chemokine C-C ligand (CCL)-2 and -8, monocyte chemotactic protein-1 and -2, interleukin-(IL)-16, RANTES and IL-8 [3], [4]. The contribution of fibroblasts to the pathogenesis of autoimmune diseases, such as rheumatoid arthritis and Graves’ disease, has been recently recognized [5], [6].

Excess fibrogenesis in CF is not understood and may represent a target therapy. We hypothesized that fibroblast dysfunctions represent a major characteristic of CF. To test this new hypothesis, we investigated the phenotype of lung and skin fibroblasts from mice homozygous for F508del, the most common CFTR mutation [7]. Markers of inflammatory and fibrotic responses, and pathology studies in lungs and skin were analyzed in *in vivo* models of bleomycin-induced fibrosis. Cell proliferation and differentiation into myofibroblasts, a specialized type of fibroblasts activated during wound healing, and expression of inflammatory mediators were investigated in purified cultured lung and skin fibroblasts. We also analyzed whether these responses are influenced by vardenafil, a clinically approved cGMP-dependent phosphodiesterase type 5 inhibitor (PDE5i). Vardenafil was tested based on its potential application in CF: we have previously shown that it is able to increase defective F508del-CFTR dependent chloride transport across the mouse nasal mucosa [8], [9] and to prevent inflammation [10]. In this work we show, for the first time, that CF fibroblasts display an altered phenotype with increased proliferation and myofibroblast differentiation, higher sensitivity to growth factors and overresponses of proinflammatory and fibrotic mediators. Vardenafil prevents dysregulated fibroblast responses; this highlights its potential in CF pharmacotherapy.

## Methods

### Animal Models

Adult female 129/FVB *Cftr^tm1EUR^* mice homozygous for the F508del mutation [7] and C57Bl6 *Cftr* ^UNC^ knockout mice were housed under conventional conditions [11]. Bleomycin (Sanofi, Diegem, Belgium) and vardenafil HCl (Bayer, West Haven, Germany), prepared in saline, were administered by pharyngeal aspiration into the lungs, topical skin injection or intraperitoneal injection. The experimental protocol was approved by the local ethical committee for animal research at the Université catholique de Louvain (2010/UCL/MD/034) and conformed to the European Community regulations (CEE n° 86/609).

### Bronchoalveolar Lavage (BAL) and Lung Histology

BAL was performed as described [12]. Unlavaged lungs were inflated with 3.6% paraformaldehyde for pathology studies. Paraffin-embedded 5-µm sections were stained with hematoxylin and eosin or Masson’s Trichrome or were impregnated with silver.

### Collagen and Cytokines

Collagen was measured as described [13]. Mouse CCL-2, IL-6, transforming growth factor (TGF)-β1 and tissue inhibitor of metalloproteinase (TIMP)-1 were assessed by ELISA (R&D Systems, Abingdon, UK); limits of detection were 1.9, 3.9, 3.9 and 7.8 mg/ml, respectively.

### Cell Cultures

Perfused lungs and skin explants were digested with Liberase TH (4 mg/lung; 1.3 mg/skin explant; Roche, Vilvoorde, Belgium) and DNAse (250 µg/lung; 83 µg/skin explant; Gestimed, Brussels, Belgium) and cultured in DMEM with 10% FBS (Life technologies, Gent, Belgium) [14]. Proliferation was estimated by ^3^H-thymidine (Amersham, Gent, Belgium) incorporation or daily counting of cultured cells. Inflammatory challenging of fibroblasts was performed with 0.1 µg/ml lipopolysaccharide from *Pseudomonas aeruginosa* (LPS; Sigma Aldrich, Diegem, Belgium); 20 ng/ml mouse recombinant IL-1β; LPS *plus* 0.1 µg/ml mouse recombinant interferon (IFN)-γ or IL-4 *plus* IL-13 (10 ng/ml of each). Vardenafil (0.1 to 50 µM) was added to fibroblast cultures. Protocols for culturing nasal epithelial cells, alveolar and peritoneal macrophages are detailed in Methods S1.

### Flow Cytometry

Fluorescent surface labelling of fibroblasts were performed using antibodies against α-smooth muscle actin (SMA, clone 1A4; Sigma Aldrich), type I collagen (clone M19; Santa Cruz, Heidelberg, Germany), CD45 (clone 30-F11; BD Biosciences, Erembodegem, Belgium) and CD11c (clone HL3; BD Biosciences). Fc receptors were blocked with anti-CD16/32 (clone 2.4G2, BD Biosciences) to reduce nonspecific binding. Samples fixed in 1.25% paraformaldehyde were analyzed using FlowJo software (Ashland, OR, USA).

### Quantitative RT-PCR

RNA, extracted with Tripure®Reagent (Roche, Vilvoorde, Belgium), was reverse transcribed and resulting cDNA was used as a template in subsequent RT-PCR analysis. Sequences of interest were amplified using the forward and reverse primers (Table S1).

### Immunoprecipitation

Immunoprecipitation was performed in fibroblast lysates after incubation with mouse anti-CFTR antibody clone 24-1 (R&D Systems) coupled with G protein-conjugated magnetic Dynabeads (Invitrogen, Merelbeke, Belgium). CFTR was detected on Western blots using an Odyssey LI-COR platform (Lincoln, NE, USA).

### Immunostaining

Immunostaining of CFTR was performed in fibroblasts grown on collagen-coated cover glasses using a mouse anti-CFTR (clone 24-1) and an anti-mouse AlexaFluor 488 secondary antibody (Life technologies). Images obtained by an AxioImager microscope were processed using AxioVision Release 4.8.2.0 software.

### Statistics

Between-group comparisons were performed by ANOVA (GraphPad InStat; San Diego, CA, USA). Posthoc comparisons were made using Student’s *t* test or Tukey-Kramer HSD test, as adequate. Null hypothesis was rejected at *P*<0.05.

## Results

### Exaggerated CF Lung Responses to Bleomycin

**Figure 1:**
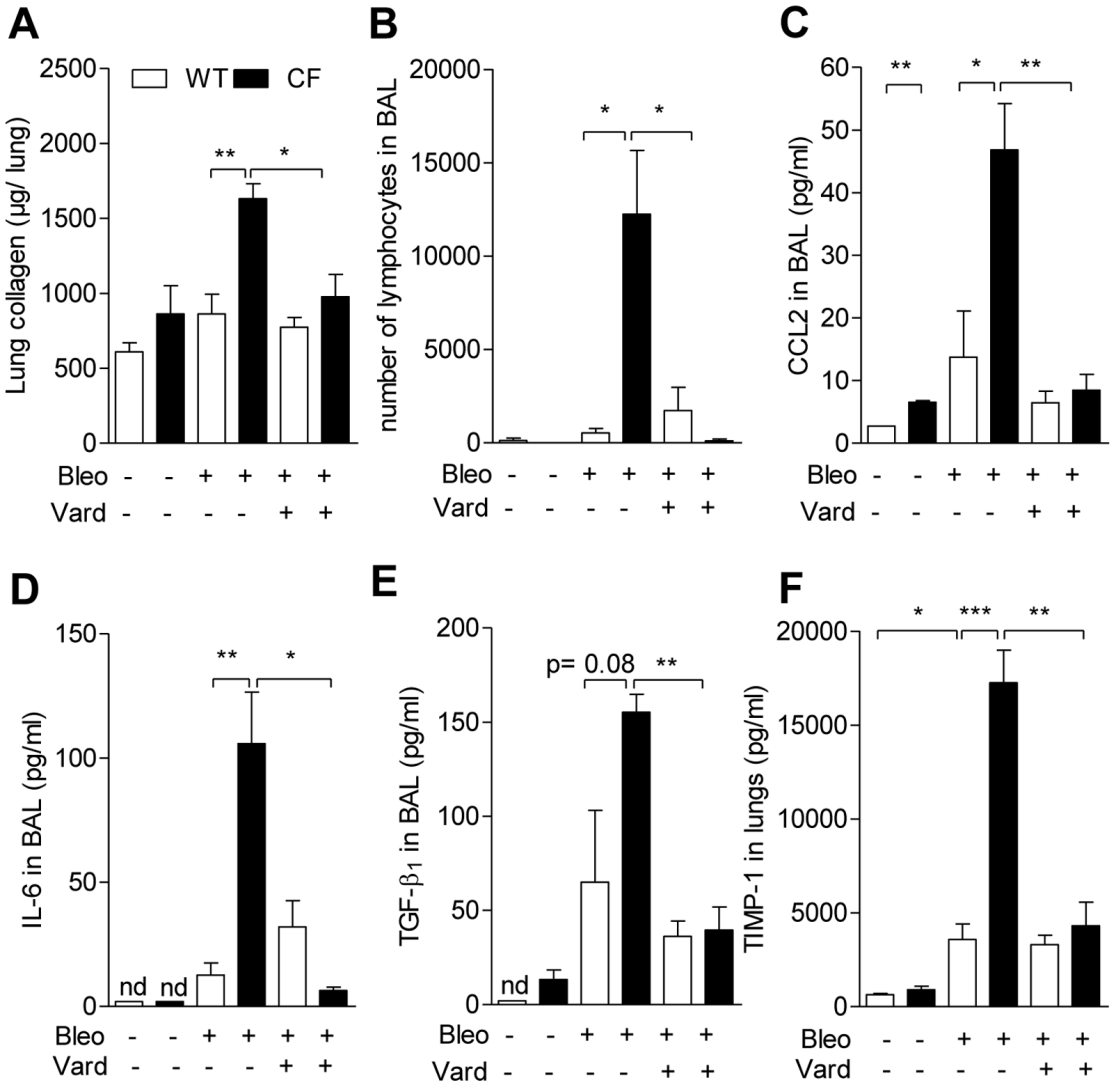
Exaggerated CF lung responses to bleomycin are attenuated by vardenafil. a) Soluble collagen content in homogenized unlavaged lungs; b) lymphocyte counts, c) CCL-2, d) IL-6, and e) TGF-β1 in bronchoalveolar lavage (BAL); and f) TIMP-1 in homogenized unlavaged lungs from CF mice homozygous for the F508del mutation and from wild-type (WT) mice 10 days after deposition into the lungs by pharyngeal aspiration of a single dose of 0.015 U bleomycin (Bleo). In case of simultaneous treatment with bleomycin and vardenafil, animals were given a first intraperitoneal injection of 0.14 mg/kg vardenafil (Vard) on the day before the bleomycin dose and every day thereafter until the day before sampling. Values are means ± SEM of 5 animals per group from a representative experiment selected from a series of 3 experiments with similar results. *: *P*<0.05; **: *P*<0.01; *** *P*<0.001 for comparison of mean values.

**Figure 2:**
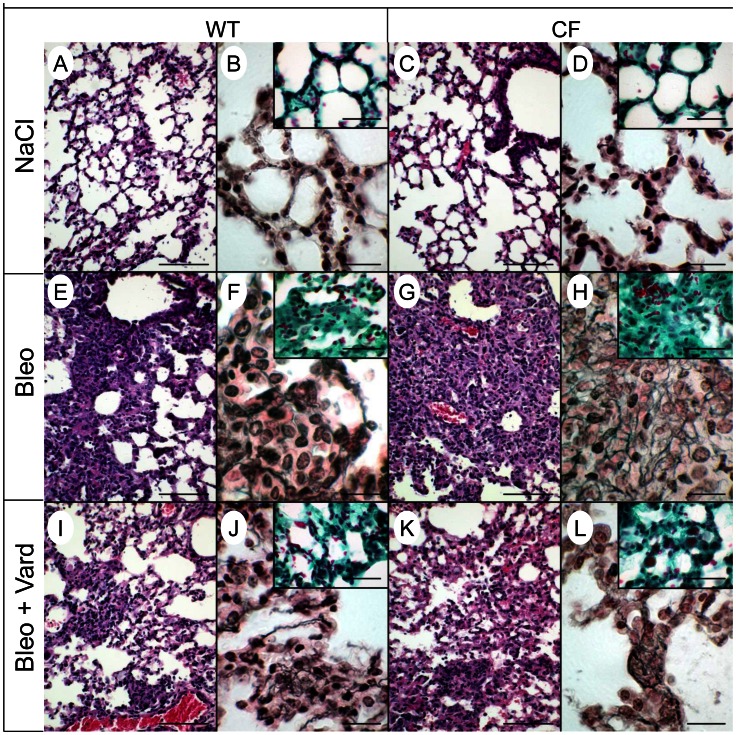
Lung histological responses to bleomycin are attenuated by vardenafil. Lung histological sections of wild-type (a,b,e,f,i,j) and CF mice homozygous for the F508del mutation (c,d,g,h,k,l) 10 days after treatment with saline (NaCl; a–d), bleomycin (Bleo; e–h) or bleomycin and vardenafil (Bleo+Vard; i–l) were stained with hematoxylin and eosin (a,c,e,g,i,k); impregnated with silver (b,d,f,h,j,l) or stained with Masson’s trichrome (inserts). Bars, 100 µm in panels a,c,e,g,i,k; 20 µm in panels b,d,f,h,j,l; and 40 µm in inserts. Representative micrographs from 5 mice per group.

To test whether fibrosis is affected in CF lungs, we applied an *in vivo* mouse model of pulmonary fibrosis induced by bleomycin [12], a glycopeptide antibiotic commonly used as cancer chemotherapy. Under control conditions, apart from CCL-2 levels which were twice as high in BAL of CF compared to wild-type mice (Figure 1c), no genotype-related differences were detected (Figure 1). After bleomycin (0.015 U per mouse), unexpectedly high (>90%) mortality was observed in CF but not in wild-type animals, that survived up to at least day 21. At day 10, the last day at which no mortality had still been observed, the magnitude of responses to bleomycin differed with genotype. In the wild-type group, profibrotic mediators, TGF-β1 and TIMP-1 were increased after bleomycin compared to the levels monitored in naive conditions; changes after challenge were more pronounced in CF (Figure 1e,f). Collagen content in whole lung homogenates was about twice as high in bleomycin-treated CF animals as in any other group (Figure 1a). Lymphocyte (Figure 1b) and neutrophil (data not shown) infiltration was higher in bleomycin-treated CF mice. Bleomycin-induced release of CCL-2 and IL-6 into BAL were three times larger in CF than in non-CF mice (Figure 1c,d). TGF-β1 and TIMP-1 were 2- and 4-fold larger in CF than in wild-type mice (Figure 1e,f). Bleomycin induced marked lung morphological changes (Figure 2). Alveolar spaces were obliterated by accumulation of fibroblasts and inflammatory cells, together with collagen deposition (Figure 2f,h inserts). Changes were more prominent in CF mice, in particular deposition of collagen III-rich argyrophilic fibres in areas of tissue condensation (Figure 2f,h).

To test whether vardenafil modulates bleomycin-induced responses, animals were treated with this PDE5i selected because, compared to sildenafil, it is more soluble, more potent and has a longer lasting effect on activation of F508del-CFTR protein [8], [9]. Vardenafil treatment lowered CF overreactions down to the same level as in wild-type, no significant changes being observed in the wild-type group (Figure 1a–f). Vardenafil reduced extent and severity of histopathological inflammatory and fibrotic transformations in CF and wild-type lung sections of mice treated with bleomycin (Figure 2i–l). The data indicate that F508del-CFTR mutation is associated with exaggerated airway inflammatory and fibroproliferative repair processes that can be prevented by treatment with vardenafil.

### Mouse Fibroblasts Express CFTR Protein

**Figure 3:**
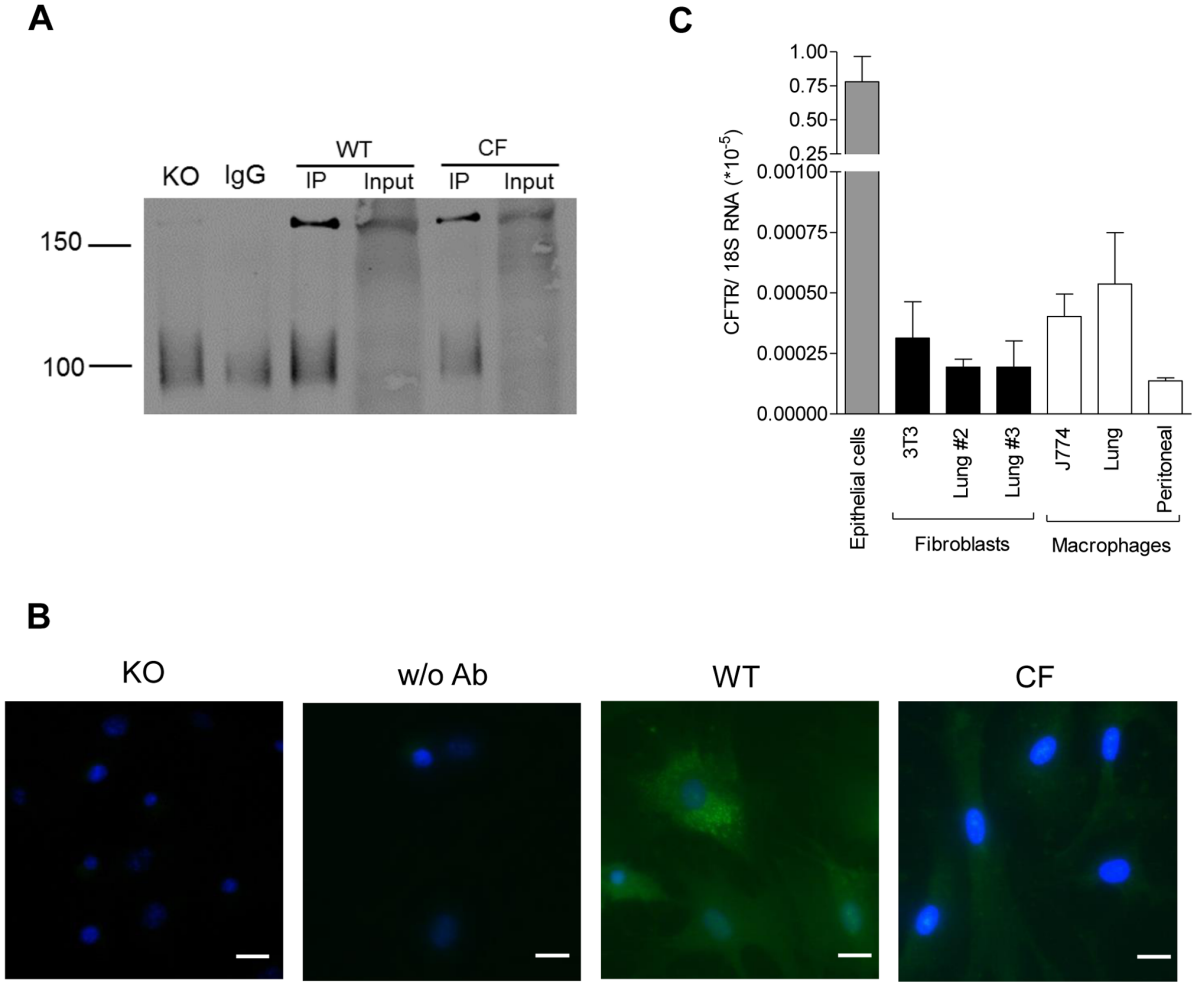
Mouse fibroblasts express CFTR protein. CFTR expression in mouse nasal epithelial cells in primary cultures, fibroblasts (3T3 cell line and lung cells at passages #2 and #3) and macrophages (J774 cell line and alveolar and peritoneal cells in primary cultures). a) Immunoblots of precipitates for IgG blank and lung fibroblasts from *Cftr* knockout mice (KO), wild-type (WT) and CF mice homozygous for the F508del mutation. Immunoprecipitates (IP) using 4×10^6^ fibroblasts grown in Petri dishes were lysed in a buffer (25 mM Tris-HCl pH 7.5, 150 mM NaCl, 1% Triton X-100) supplemented with Complete PIC (Roche) and incubated with mouse anti-CFTR antibody clone 24-1 coupled with G protein-conjugated magnetic Dynabeads. Data selected from at least 4 experiments with similar results. As expected for mouse CFTR, a band was recognized at 160 kDa but not detected when IPs were performed with non-immune IgG. b) Immunofluorescence labelling for CFTR (green) in fibroblasts purified from *Cftr* knockout (KO) mice, wild-type mice with (WT) or without CFTR antibody (w/o Ab), and CF mice homozygous for the F508del mutation. Fibroblasts grown on collagen-coated cover glasses were fixed with acetone and permeabilized with 0.25% Triton X-100. Detection was obtained using anti-mouse AlexaFluor 488 secondary antibody after overnight incubation with anti-CFTR antibody clone 24-1. Nuclei stained with DAPI blue. Bars: 20 µm. Data selected from at least 3 experiments with similar results. c) Total CFTR mRNA expression, using 18S rRNA as a reference. Data expressed as means ± SEM of 3–8 samples per group.

Before testing the hypothesis that F508del-CFTR is associated with altered fibroblastic functions, we first examined CFTR expression in purified fibroblasts from wild-type lungs. Epithelial cells strongly express CFTR. Macrophages express CFTR at a low but functional level [15]. A functionally altered [16], [17] but controversial [18], [19] phenotype has been recognized in dermal fibroblasts from CF patients. To investigate CFTR protein and mRNA expression, comparative analyses were performed in mouse fibroblasts, macrophages and epithelial cells from different origins. Lower CFTR protein expression was found in F508del-CF than in wild-type fibroblasts; fibroblasts isolated from *Cftr* knockout mice showed no detectable expression (Figure 3a,b). CFTR mRNA expression in wild-type lung fibroblasts showed levels similar to those found in 3T3 cell line. Cell passage did not seem to affect CFTR expression as quite similar protein expression levels were observed at the second and the third passages (Figure 3c). Similar levels were also observed in J774 cells, in alveolar and peritoneal macrophages. Predictably, CFTR was highly expressed in airway epithelial cells (Figure 3c; Methods S1). These data indicate that similar CFTR expression is found in fibroblasts as in other non-epithelial cells, such as macrophages.

### Proliferation and Myofibroblast Differentiation of CF Lung Fibroblasts

All experiments using cultured fibroblasts were performed at the second passage when 86% of cultured cells were negative for CD45 leukocyte-specific cell-surface marker, more than 40% of cells were positive for type I collagen and α-SMA, and less than 3% CD11c positive cells, indicating macrophage contamination, were found (data not shown).

**Figure 4:**
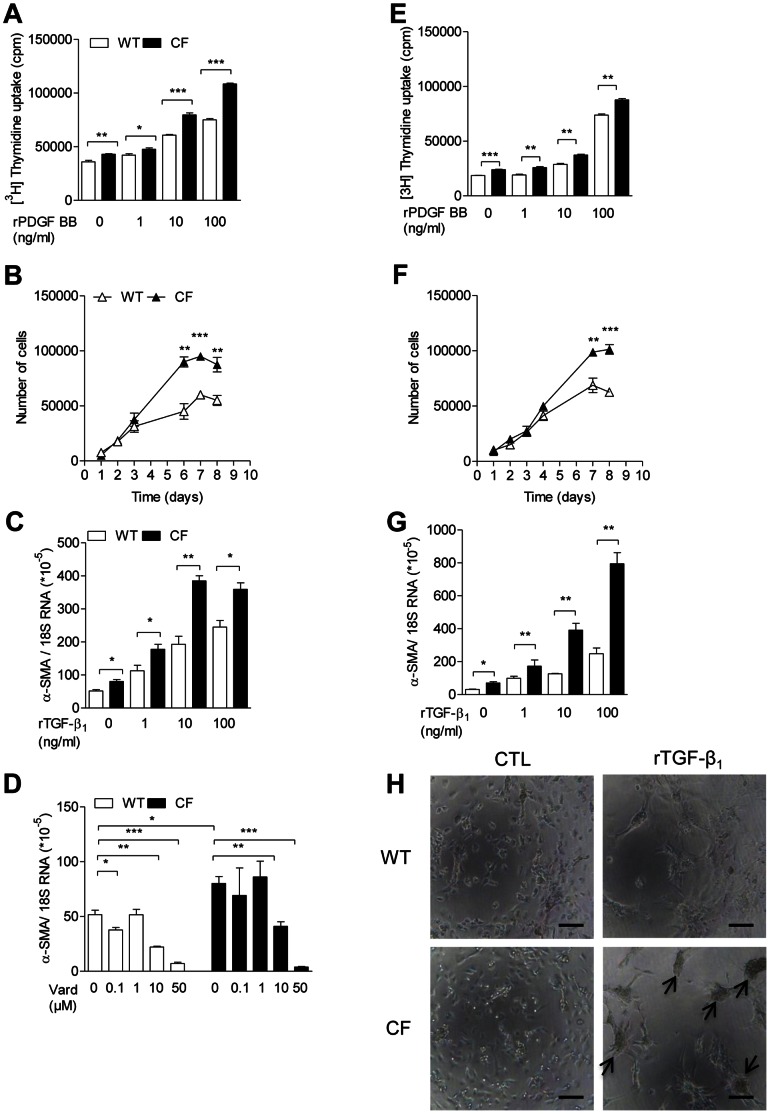
Exaggerated proliferation and myofibroblast differentiation of CF fibroblasts. Cell proliferation and myofibroblast differentiation in cultured lung (A–D) and skin (E–H) fibroblasts at the second passage purified from CF mice homozygous for the F508del mutation and from wild-type (WT) mice. A,e) Uptake of^ 3^H-thymidine (1 µCi/well) assessed in cultured cells seeded at 30×10^3^ cells/well, in the absence of any added growth factor to culture media or in the presence of 1 to 100 ng/ml human rPDGF-BB for 1 h. After 48 h, adherent cells were trypsinized before ^3^H-thymidine counting. Data expressed as counts per minute (cpm). b,f) Cell growth analysis assessed by daily counting, in a Neubauer chamber, of trypsinized cells cultured in the absence of any added growth factor to culture media. c,g) α-SMA mRNA expression, using 18S RNA as a reference, assessed in the absence of any added growth factor to culture media or in the presence of 1 to 100 ng/ml human rTGF-β1 for 24 h. d) α-SMA mRNA expression assessed in the presence of 0.1 to 50 µM vardenafil (Vard) for 6 h. h) Micrographs of fibroblast cultures under stimulation with 10 ng/ml human rTGF-β1. Arrows identify formation of cellular aggregates. Bars: 100 µm. Values are means ± SEM of 3 multi(96)well cultures per group from a representative experiment selected from at least 3 experiments with similar results. *: *P*<0.05; **: *P*<0.01; *** *P*<0.001 for comparison of mean values.

To test the hypothesis that F508del-CFTR mutation is associated with altered fibroblastic functions, we examined cell proliferation and myofibroblast differentiation in purified cultured fibroblasts from lungs of CF and wild-type mice. ^3^H-thymidine incorporation was higher in CF than in wild-type fibroblasts, even in the absence of any added growth factor (Figure 4a). In the presence of human recombinant platelet-derived growth factor (rPDGF)-BB, thymidine incorporation increased in a dose-dependent manner, reaching higher values in CF fibroblasts (Figure 4a). Cell growth curve analysis, assessed by daily counting of trypsinized cells in culture, showed higher density at a plateau phase in CF fibroblasts (Figure 4b). Expression of α-SMA, a marker of differentiation towards a myofibroblast phenotype, was higher in CF fibroblasts, even in naive conditions (Figure 4c,d). The stimulating effect of human rTGF-β1 on α-SMA expression was increased in CF compared to wild-type fibroblasts (Figure 4c). Vardenafil did not modify cell growth (data not shown) but decreased α-SMA mRNA expression in both CF and non-CF fibroblasts (Figure 4d). In the presence of 50 µM vardenafil, α-SMA expression in CF fibroblasts reached levels as low as those monitored in similarly treated wild-type cells. These findings indicate that CF lung fibroblasts display a modified extracellular matrix producing phenotype in naive conditions and under stimulation by growth factors, and that vardenafil inhibits this phenotype in CF and in non-CF lung fibroblasts.

### Dysregulated Fibrogenic Processes in CF are not Solely Confined to Lung Fibroblasts

To test whether altered fibroblast properties are confined to lung fibroblasts, we examined histopathology in response to bleomycin, fibroblast proliferation and myofibroblast differentiation in another tissue. Histological analyses of skin lesions after topical application of bleomycin (Methods S1) confirmed increased neofibrogenesis in CF animals with a dermis containing more numerous inflammatory cells and collagen III-rich argyrophilic fibres, whereas bleomycin-challenged wild-type tissues were more scarred (Figure S1). In addition, epidermal layer and hair follicles were hyperplastic. The changes were reduced in vardenafil-treated animals.

Cultured CF dermal fibroblasts showed, as observed in CF lung fibroblasts, increased proliferation (Figure 4e,f) and differentiation into myofibroblasts (Figure 4g). As for lung fibroblasts (Figure 4c,d), baseline α-SMA overexpression and TGF-β1 hypersensitivity were noted in CF skin fibroblasts (Figure 4g). The exacerbated capacity of CF cells to differentiate into myofibroblasts was also illustrated by formation, in CF cultures, of cellular aggregates indicating cell contracture activity (Figure 4h). These findings indicate that dysregulated fibrogenic processes in CF are not solely confined to lung fibroblasts.

### Proinflammatory Status of CF Fibroblasts and Preventing Effect of Vardenafil

**Figure 5:**
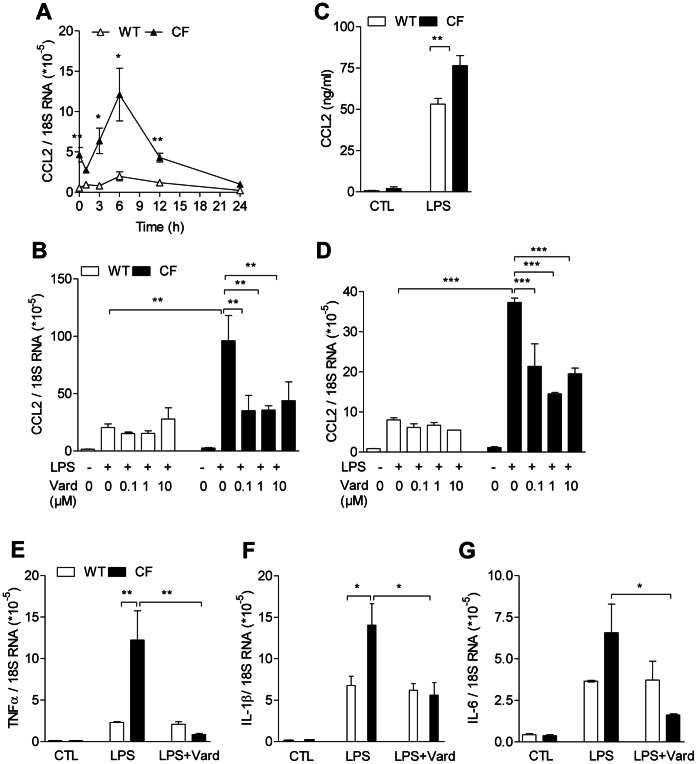
Vardenafil prevents overresponsive proinflammatory status in CF fibroblasts. a,b–g) mRNA and c) protein expression of proinflammatory cytokines in response to 0.1 mg/ml LPS in lung (a,b,e–g) and skin (c,d) cultured fibroblasts at the second passage purified from CF mice homozygous for the F508del mutation and from wild-type (WT) mice. At the mRNA level, markers were assessed 3 h after LPS stimulation. At the protein level, CCL-2 (c) was assessed 24 h after LPS stimulation. Vardenafil (Vard; 0.1 µM) was used for TNF-α (e), IL-1β (f) and IL-6 (g) mRNA expression studies. 18S RNA used as a reference gene. Values are means ± SEM of 3 multi(96)well cultures per group from a representative experiment selected from at least 3 experiments with similar results. *: *P*<0.05; **: *P*<0.01; *** *P*<0.001 for comparison of mean values.

We then investigated whether fibroblasts play a role in the exaggerated inflammatory responses in CF. Under non-stimulated conditions, CCL-2 mRNA expression was enhanced in CF lung fibroblasts (Figure 5a). After stimulation with LPS, kinetics of the transcript showed higher levels in CF lung fibroblasts peaking 6 h after stimulation (Figure 5a). LPS-induced CCL-2 transcript was also increased in CF compared to wild-type skin fibroblasts (Figure 5d). Accordingly, LPS-induced CCL-2 protein release was larger in CF than in wild-type dermal fibroblasts (Figure 5c). Responses to LPS of other proinflammatory markers, such as TNF-α, IL-1β and IL-6, were increased in CF compared to non-CF lung fibroblasts (Figure 5e–g). CF overresponses were also observed under the influence of other proinflammatory stimuli, such as mouse rIL-1β (Figure S2a). Moreover, responses of TNF-α and inducible nitric oxide synthase (iNOS) driven by a F1 polarization protocol, consisting on a combination of LPS *plus* IFN-γ were increased in CF compared to wild-type lung fibroblasts (Figure S2b,c). However, the anti-inflammatory Ym1-2 chitinase-like protein after stimulation with a F2 protocol, consisting in the combination of IL-4 *plus* IL-13 stimuli, did not show any difference between CF and non-CF cells (Figure S2d).

To test whether vardenafil reduces proinflammatory responses in CF and non-CF lung and dermal fibroblasts, we first assessed CCL-2 transcripts after treatment with the PDE5i. The LPS-stimulating effect was inhibited by 0.1 µM vardenafil, no additional inhibitory effect being observed with larger concentrations (Figure 5b,d). Vardenafil (0.1 µM) also downregulated TNF-α, IL-1β and IL-6 mRNA expression in CF lung fibroblasts, with no effect in non-mutated cells. These findings indicate that CF fibroblasts from different tissue origins display an overresponsive proinflammatory status and that vardenafil prevents fibroblast inflammatory responses.

## Discussion

The present study was designed to test the hypothesis that fibroblasts represent master cells in fibrogenesis in CF. Clinical studies have suggested that uncontrolled airway remodelling in CF begins early in life [2], [20]. Ultrastructural changes of airway scarring, including thickening of epithelial basement membrane and collagenisation of lamina propria, have been linked to a worse pulmonary outcome [21]. Additional evidence emerges from the demonstration that overexpression of TGF-β1, a key growth factor for fibroblast functions, is associated with a more severe CF lung phenotype [22]. Studies on polymorphisms of *TGF-β1* have given rise to the suggestion that the gene is a modifier of CF lung function [23]. However, mediators of fibrosis events in CF are not understood.

We show an increased release into CF lungs of TGF-β1, a multi-functional protein involved in wound repair, myofibroblast differentiation and synthesis of several components of connective tissue matrix [24]. In CF lungs challenged with bleomycin, overexpression of TIMP-1, an early and predictive marker of tissue remodelling [25], reflects a protease/antiprotease imbalance associated with tissue damage. Increased basal CCL-2 expression in CF mice is in line with the increased levels we have previously demonstrated in the bronchoalveolar and peritoneal compartments of CF mice [13]. The enhanced lymphocyte and neutrophil lung infiltrates denote that fibrosis in CF involves multiple cell types. CF mice challenged with bleomycin display exaggerated proinflammatory and matrix accumulation responses. Histological changes in lungs and skin included increased inflammatory infiltration and matrix neoformation with reticulinic fibres accumulation. Accordingly, excessive skin fibrosis and epidermal thickening have been reported in F508del-CF mice during mite infestation [26].

The assumption that function of fibroblasts is solely related to their capacity to produce extracellular matrix has been revisited, based on recent demonstrations of their critical roles in pathogenesis of autoimmune diseases [5], [6]. However, the exact role of fibroblasts in inflammation has not been fully clarified. Data presented here clearly show, for the first time, that purified cultured fibroblasts from CF tissues have an altered phenotype. CF fibroblasts proliferate exaggeratedly, overreact to PDGF-BB and TGF-β1 and display increased myofibroblat differentiation with neoexpression of α-SMA, the actin isoform related to contractile activity in vascular smooth muscle cells [24]. This work confirms CCL-2 dysregulation in CF immune responses [13] and suggests that different cell populations contribute to chemokine overproduction in CF: we have previously shown that CF alveolar and peritoneal macrophages [13], but not CF respiratory epithelial cells [27], produce manifold larger amounts of CCL-2 than non-CF cells. Overproduction of multiple proinflammatory mediators (CCL-2, TNF-α, IL-1β, IL-6), triggered by different stimuli (IL-1β, LPS and IFN-γ) in fibroblasts from different (lung and dermal) origins, denotes that the proinflammatory status in CF fibroblasts is a ubiquitous, extensive and complex process involving multiple signalling pathways and transcription factors that may act separately or in concert.

CFTR expression in CF fibroblasts has not been thoroughly investigated. It has been observed, using northern analysis, in non-epithelial cells including lung fibroblasts, monocytes and alveolar macrophages [28]. Our original findings indicate that CFTR mRNA and protein are expressed in lung fibroblasts at levels similar to those observed in macrophages. Dysregulated fibroblasts can contribute to the pathogenesis of CF and may highlight fibroblasts as a target for development of novel therapeutic strategies. As CF dermal fibroblasts reproduce the basic dysfunctional properties observed in lung fibroblasts, and because, unlike lungs, skin is readily available and shows no secondary changes, such as chronic infection and mucus obstruction, cultured fibroblasts explanted from skin biopsies of CF patients could represent a prime choice tissue to search for the primary defect and to test efficacy of therapeutic strategies.

Our data show that vardenafil reduces overexpression of proinflammatory and profibrotic markers, arguing that fibroblasts represent target cells of the drug. While submicromolar concentrations appeared to influence proinflammatory and profibrotic responses, much larger (10 µM) drug concentrations seemed to be required to modulate myofibroblast differentiation with α-SMA neoformation. The 0.1 µM concentration is clinically relevant because it corresponds roughly to plasma levels measured 1 h after administration of an oral therapeutic dose of 20 mg vardenafil to healthy volunteers. Our data are consistent with a previous report showing that sildenafil, a vardenafil analogue, reduces fibrosis in the *mdx* mouse model of Duchenne muscular dystrophy [29]: treating *mdx* mice with sildenafil reduced endomysial fibrosis and TNF-α overexpression in diaphragm. In line with a recent paper [30], our study highlights the potentiality of vardenafil to treat CF: beside its ability to normalize the basic transepithelial chloride transport defect [8], [9], it modulates inflammation [10] and fibrogenesis in CF.

This work provides novel insights into the contribution of fibroblasts to the pathogenesis of CF. Fibroblasts could represent a potentially attractive target for future trials and possible treatments in CF. Moreover, this study provides compelling new support for targeting cGMP signalling pathway in CF pharmacotherapy.

## Supporting Information

Figure S1 **Exaggerated inflammatory and fibrotic responses to bleomycin and preventing effect of vardenafil in skin of CF mice.** Histological sections of skin from wild-type (a,b,e,f,i,j) and CF (c,d,g,h,k,l) mice homozygous for the F508del mutation 21 days after treatement with saline (NaCl; a–d), bleomycin (Bleo; e–h) or bleomycin and vardenafil (Bleo+Vard; i–l) were stained with hematoxylin and eosin (a,c,e,g,i,k) or with Masson’s trichrome (b,d,f,h,j,l). Representative micrographs from 3–4 mice per group. Bars correspond to 100 µm.
(PDF)

Figure S2 **Extensive overproduction of inflammatory mediators by CF lung fibroblasts.** Responses of pro- and anti-inflammatory markers to *Pseudomonas aeruginosa* lipopolysaccharide (LPS, 0.1 mg/ml) or to F1/F2 stimulation in lung cultured fibroblasts at the second passage purified from CF mice homozygous for the F508del mutation and from wild-type (WT) mice. a) CCL-2 protein assessed by ELISA, 24 h after stimulation with 20 ng/ml mouse recombinant IL-1β stimulation. (b,c) TNF-α, iNOS mRNA expression assessed 3 h after F1 polarization induced by adding 0.1 mg/ml LPS *plus* 0.1µg/ml mouse recombinant IFN-γ. 18S RNA was used as a reference gene. (d) Ym1-2 anti-inflammatory marker mRNA expression 3 h after F2 polarization induced by adding IL-4 *plus* IL-13 (10 ng/ml of each). 18S RNA was used as a reference gene. Values are means ± SEM of 3 multi(96)well cultures per group from a representative experiment selected from at least 3 experiments with similar results. *: *P*<0.05; **: *P*<0.01; *** *P*<0.001 for comparison of mean values.
(PDF)

Methods S1 The detailed methods of cell cultures and skin fibrosis model are proposed in Methods S1.
(DOC)

Table S1 Depicts the sequences of forward and reverse primers used to perform quantitative RT-qPCR.
(DOC)
