# Supplementary figures and images for: Dysregulated Proinflammatory and Fibrogenic Phenotype of Fibroblasts in Cystic Fibrosis
Source: PLoS One. 2013 May 29;8(5):e64341. doi: 10.1371/journal.pone.0064341 (PMC3667188; doi:10.1371/journal.pone.0064341)

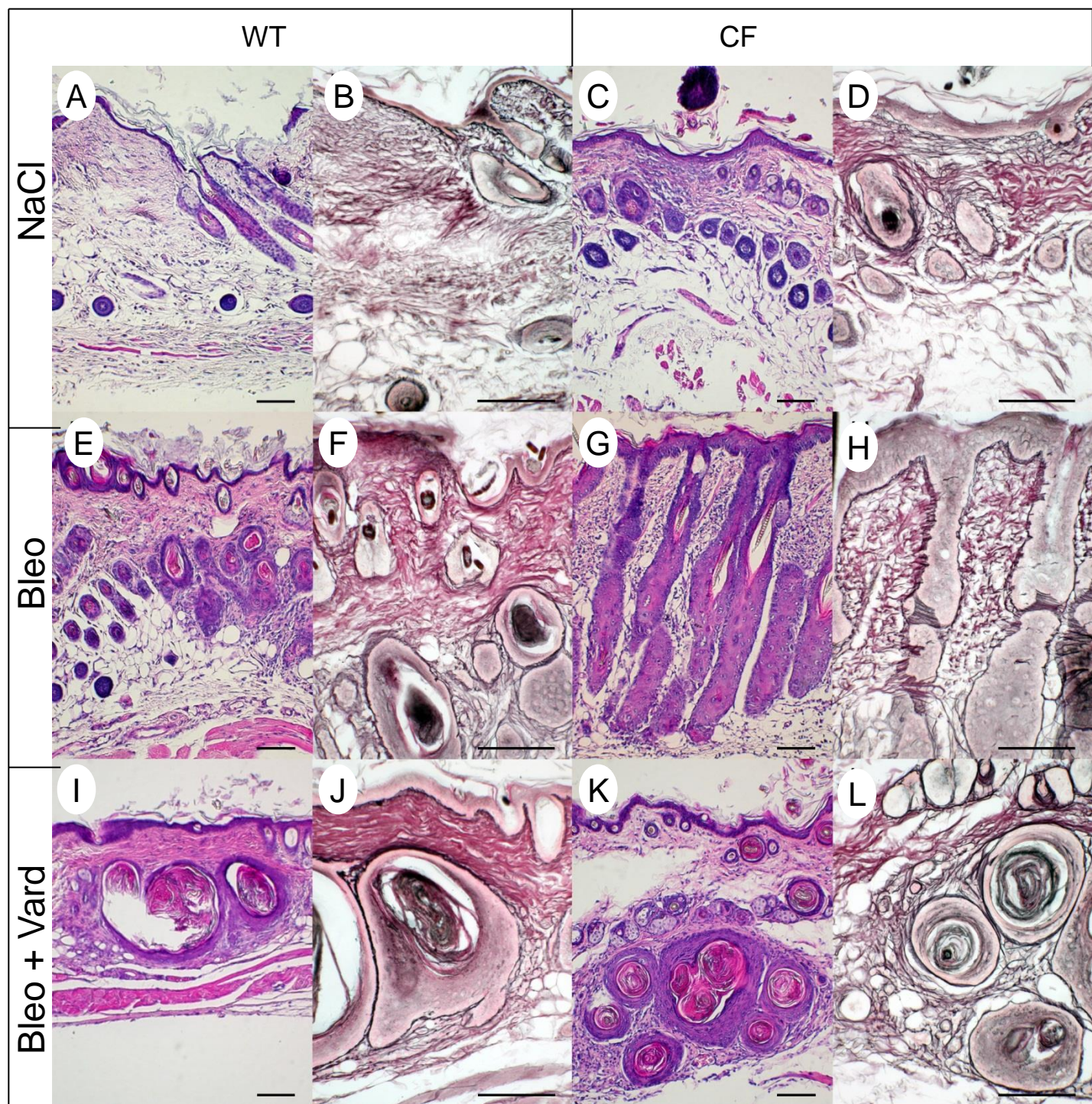

Supplement: Figure S1 — Exaggerated inflammatory and fibrotic responses to bleomycin and preventing effect of vardenafil in skin of CF mice. Histological sections of skin from wild-type (a,b,e,f,i,j) and CF (c,d,g,h,k,l) mice homozygous for the F508del mutation 21 days after treatement with saline (NaCl; a–d), bleomycin (Bleo; e–h) or bleomycin and vardenafil (Bleo+Vard; i–l) were stained with hematoxylin and eosin (a,c,e,g,i,k) or with Masson’s trichrome (b,d,f,h,j,l). Representative micrographs from 3–4 mice per group. Bars correspond to 100 µm. (PDF) [file pone.0064341.s001.pdf]

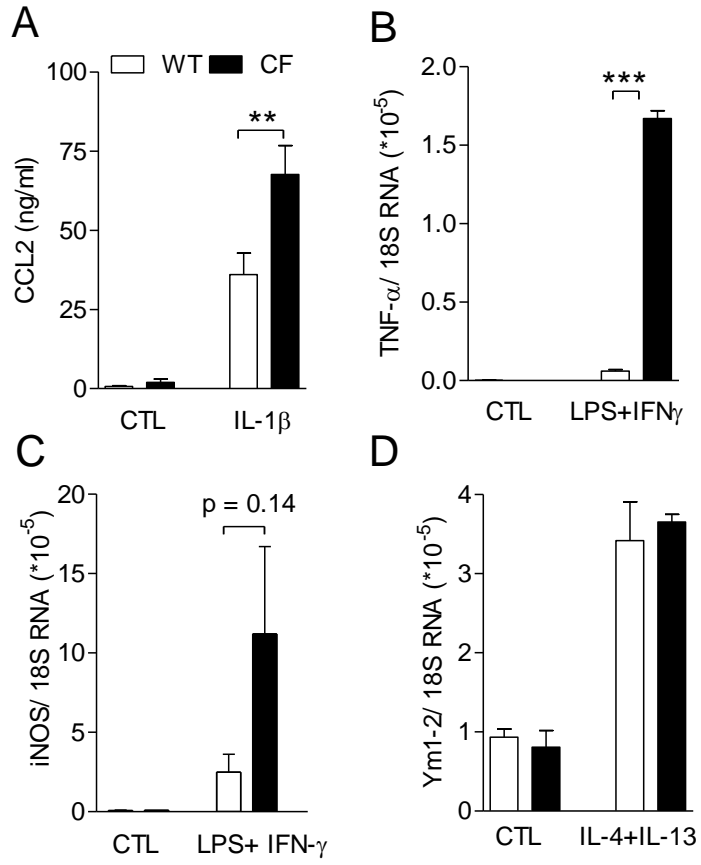

Supplement: Figure S2 — Extensive overproduction of inflammatory mediators by CF lung fibroblasts. Responses of pro- and anti-inflammatory markers to Pseudomonas aeruginosa lipopolysaccharide (LPS, 0.1 mg/ml) or to F1/F2 stimulation in lung cultured fibroblasts at the second passage purified from CF mice homozygous for the F508del mutation and from wild-type (WT) mice. a) CCL-2 protein assessed by ELISA, 24 h after stimulation with 20 ng/ml mouse recombinant IL-1β stimulation. (b,c) TNF-α, iNOS mRNA expression assessed 3 h after F1 polarization induced by adding 0.1 mg/ml LPS plus 0.1µg/ml mouse recombinant IFN-γ. 18S RNA was used as a reference gene. (d) Ym1-2 anti-inflammatory marker mRNA expression 3 h after F2 polarization induced by adding IL-4 plus IL-13 (10 ng/ml of each). 18S RNA was used as a reference gene. Values are means ± SEM of 3 multi(96)well cultures per group from a representative experiment selected from at least 3 experiments with similar results. *: P<0.05; **: P<0.01; *** P<0.001 for comparison of mean values. (PDF) [file pone.0064341.s002.pdf]
