# Supplementary material for: Dysregulated Proinflammatory and Fibrogenic Phenotype of Fibroblasts in Cystic Fibrosis
Source: PLoS One. 2013 May 29;8(5):e64341. doi: 10.1371/journal.pone.0064341 (PMC3667188; doi:10.1371/journal.pone.0064341)
Supplement: Methods S1 — The detailed methods of cell cultures and skin fibrosis model are proposed in Methods S1. (DOC) [file pone.0064341.s003.doc]

**Methods S1**

**Cell cultures**

Primary cultures of resident peritoneal and alveolar macrophages from CF mice homozygous for the F508del mutation and wild-type mice were obtained from peritoneal cavity lavage (PCL) and bronchoalveolar lavage (BAL) from the same group of mice as previously reported [1]. Briefly, animals were killed by subcutaneous injection of 24 mg pentobarbital (Certa, Braine-l’Alleud, Belgium), the peritoneal cavity was then lavaged with 10ml of sterile 0.9% NaCl. BAL was performed by cannulating the trachea and lavaging the lungs four times with 1 ml of sterile 0.9% NaCl. This step was repeated with 3 additional volumes (1 ml) of sterile saline. PCL and BAL were centrifuged (280 x *g*, 10 min, 4°C). Cell pellets were resuspended in HBSS (Life technologies, Gent Belgium) to determine total and differential cell counts, performed on cytocentrifuge preparations and stained with Diff Quick (Dade, Brussels, Belgium). Supernatants were used for cytokine analyses by ELISA. Cell pellets collected by an additional centrifugation (280 x g, 10 min, 4°C) were resuspended in DMEM supplemented with 10% FBS (Life technologies). Cells were seeded at 37°C in an atmosphere of 5°C CO_2_ in 96-well culture plates at 10^5^ macrophages/well. After cell adherence (2 h), the supernatants containing non-adherent cells were removed by aspiration.

Epithelial cells were isolated and purified from nasal mucosa of CF mice homozygous for the F508del mutation and wild-type mice as previously reported [2]. Briefly, excised nasal mucosa was incubated at 37°C for 60 min in 10 ml of a digestion solution containing HBSS medium, 1% penicillin-streptomycin to which 1 mg/ml pronase (Sigma Aldrich, Diegem, Belgium) was added; under these conditions a final cell density of 2.5 x 10^5^ purified epithelial cells/mouse was obtained. At the end of pronase digestion, the cell suspension was filtered through a 70 µm cell strainer (BD Biosciences, Erembodegem, Belgium) in a 50 ml falcon tube. Cells were resuspended in 10 ml of an incubation medium composed of equal parts of DMEM and F-12 medium, spun for 10 min (120 g at 4°C) and the pellet was resuspended in 3 ml of the incubation medium. Following 2 h adherence in 6-well culture plates at 37°C in an atmosphere of 5% CO_2_, non epithelial cells, macrophages and fibroblasts, were depleted. Non-adherent cells were collected by an additional centrifugation (10 min at 120 g at 4°C) and pellets were resuspendend in BEG medium (Life technologies) to reach 2 x 10^5^ epithelial cells/ml. Cells (2 x 10^5^/100 µl) were seeded at 37°C for 24h in an atmosphere of 5% CO2 in 96-well culture plates.

3T3 fibroblasts and J774 macrophage cell line were cultured in DMEM-glutamax (Life technologies) supplemented with 10% FBS and 1% antibiotics (100 U/ml penicillin, 100 µg/ml streptomycin and 0.25 µg/ml fungizone; Life technologies).

**In vivo protocol for bleomycin-induced dermal fibrosis**

Skin fibrosis was induced in young adult F508del-CF and wild-type mice by local injections of bleomycin for 21 days [3]. Briefly, 100 µl of bleomycin dissolved in sterile saline at a concentration of 0.5 mg/ml was administered every day by subcutaneous injection into defined areas of 1 cm^2^ on the upper back. Subcutaneous injections of 100 µl of 0.9% NaCl were used as controls. Vardenafil (0.14mg/kg) was administered daily by intraperitoneal route from the day before the first bleomycin/saline injection till the day of killing by cervical dislocation. Areas of lesional skin were then excised and fixed in 3.6% buffered paraformaldehyde (Sigma Aldrich) for histopathology studies. After overnight fixation, skin pieces were embedded in paraffin, 5 µm thick sections were taken and stained with hematoxylin and eosin or impregnated with silver according to Gordon and Sweets [4].

**References**

1. Meyer M, Huaux F, Gavilanes X, van den Brûle S, Lebecque P, et al. (2009) Azithromycin reduces exaggerated cytokine production by M1 alveolar macrophages in cystic fibrosis. Am J Respir Cell Mol Biol 41:590-602.
2. Gavilanes X, Huaux F, Meyer M, Lebecque P, Marbaix E, et al. (2009) Azithromycin fails to reduce increased expression of neutrophil-related cytokines in primary-cultured epithelial cells from cystic fibrosis mice. J Cyst Fibros 8:203-210.
3. Avouac J, Fürnrohr BG, Tomcik M, Palumbo K, Zerr P, et al. (2011) Inactivation of the transcription factor STAT-4 prevents inflammation-driven fibrosis in animal models of systemic sclerosis. Arthritis Rheum 63:800-809.
4. Gordon H, Sweets HH Jr. A simple method for silver impregnation of reticulum (1936) Am J Pathol 12: 545-552.
