# Supplementary material for: Dysregulated Proinflammatory and Fibrogenic Phenotype of Fibroblasts in Cystic Fibrosis
Source: PLoS One. 2013 May 29;8(5):e64341. doi: 10.1371/journal.pone.0064341 (PMC3667188; doi:10.1371/journal.pone.0064341)
Supplement: Table S1 — Depicts the sequences of forward and reverse primers used to perform quantitative RT-qPCR. (DOC) [file pone.0064341.s004.doc]

**Table S1 – Forward and reverse primers (Invitrogen) of amplified mRNA sequences of interest**

| Gene | Forward primers | Reverse primers |
| --- | --- | --- |
| TNF-α  CCL-2  IL-1β  i-NOS  IL-6  Ym1-2  α-SMA  CFTR  18SRNA | 5’-GCC TCT TCT CAT TCC TGC TTG T-3’  5’-AGC CAG ATG CAG TTA ACG CC-3’  5’-GAC GGA CCC CAA AAG ATG AAG-3’  5’-TGA AGA AAA CCC CTT GTG CT-3’  5’-GAG GAT ACC ACT CCC AAC AGA CC -3’  5’-TGT TCT GGT GAA GGA AAT GCG-3’  5’-GGA GTA ATG GTT GGA ATG GGC-3’  5’-AAA AGA ATC CCC AGC TTA TCC AC-3’  5’-CGG CTA CCA CAT CCA AGG AA-3’ | 5’-GGC CAT TTG GGA ACT TCT CA-3’  5’-TTT GGG ACA CCT GCT GCT G-3’  5’-CTC TTG TTG ATG TGC TGC TGT G-3’  5’-TGG AAC ATT CTG TGC TGT CC-3’  5’-CAC AAC TCT TTT CTC ATT TCC ACG -3’  5’-CGT CAA TGA TTC CTG CTC CTG-3’  5’-GCC TTA GGG TTC AGT GGT GC-3’  5’-GAC AGC CTT GGT GAC TTC CC-3’  5’-ATA CGC TAT TGG AGC TGG AAT TAC C-3’ |
